# Mineral composition of elements in wood-growing mushroom species collected from of two regions of Poland

**DOI:** 10.1007/s11356-020-10788-y

**Published:** 2020-09-17

**Authors:** Mirosław Mleczek, Monika Gąsecka, Anna Budka, Marek Siwulski, Patrycja Mleczek, Zuzanna Magdziak, Sylwia Budzyńska, Przemysław Niedzielski

**Affiliations:** 1 Department of Chemistry, Poznan University of Life Sciences, Poznań, Poland; 2 Department of Mathematical and Statistical Methods, Poznan University of Life Sciences, Poznań, Poland; 3 Department of Vegetable Crops, Poznan University of Life Sciences, Poznań, Poland; 4 Department of Ecology and Environmental Protection, Poznan University of Life Sciences, Piątkowska 94c, 60-649 Poznań, Poland; 5 Faculty of Chemistry, Adam Mickiewicz University in Poznań, Poznań, Poland

**Keywords:** Accumulation, Elements, Wood-growing mushrooms, Tree species

## Abstract

The study monitored the content of 55 elements in 21 wood-growing mushroom species collected between 2013 and 2019 from Lower and Upper Silesia in Poland. Only 27 of the elements (Ag, Al, Ba, Ca, Cd, Cu, Fe, In, K, La, Mg, Mn, Na, Nd, Ni, P, Pb, Pr, Pt, Rh, Sr, Ti, Tm, V, Y, Zn, and Zr) were detected in all mushroom species, while others (As, Au, B, Be, Bi, Ce, Co, Cr, Dy, Er, Eu, Ga, Gd, Ge, Hf, Ho, Ir, Li, Lu, Mo, Os, Pb, Rb, Re, Ru, Sb, Sc, Se, Sm, Tb, Te, Th, Tl, Tm, U, and Yb) were below the limit of detection in the fruit bodies of at least one species. Wide ranges for major elements in the whole population of all the mushroom species were as follows: 15.4–470 (Ca), 6580–44,600 (K), 314–2150 (Mg), 38.0–319 (Na), and 1100–15,500 (P) mg kg^−1^ dm, respectively. The rank sum revealed that *M. giganteus* fruit bodies were the most enriched with all detectable elements, while *A. mellea* had the lowest content of the majority of elements. Mushrooms belonging to the Hymenochaetaceae family were characterized as some of the most enriched with the studied elements, while mushrooms of the Fomitopsidaceae family had the lowest content of elements. Similarities as well as differences between the obtained results and the available literature data confirm the important role of both mushroom species and the tree on which the fungus has grown.

**Electronic supplementary material:**

The online version of this article (10.1007/s11356-020-10788-y) contains supplementary material, which is available to authorized users.

## Introduction

Mushrooms are ubiquitous organisms in the natural environment; they inhabit unpolluted areas such as forest ecosystems as well as polluted urban and industrial areas (Karaman et al. 2012; Rakić et al. 2014). They represent a distinct group of living organisms characterized by significant nutritive, pharmaceutical, and ecological value (Širić et al. 2016). Large numbers of wild growing and cultivated mushrooms contain chemical compounds that are beneficial for human health. Their nutrient potential is due to a low level of calories, high content of some vitamins and micro- and macroelements as well as protein (Gargano et al. 2017). Some species belonging to *Basidiomycetes* and *Ascomycetes* are called medicinal mushrooms because of their therapeutic effect related to bioactive compounds such as polysaccharides (including β-glucans), polysaccharide–protein complex, phenols, terpenes, steroids, alkaloids, and others (de Silva et al. 2013; Duru and Cayan 2015; Gargano et al. 2017). About 130 different therapeutic functions are attributed to a broad spectrum of bioactive compounds of fungi including antitumor, antioxidant, anti-inflammatory, antibacterial, antiallergic, cardiovascular, and neuroprotective effects (Paterson and Lima 2014; Phan et al. 2015; Prasad et al. 2015; Gargano et al. 2017). Thus, they could be of great value in prophylaxis and treatment of some diseases including obesity, hyperglycemia, and high blood pressure in the adjunct treatment of cancer patients (Guillamón et al. 2010; Guggenheim et al. 2014).

Mushrooms are responsible for the breaking down of organic matter, and they have an important role in the continual changes that take place in nature (Sesli et al. 2008). Recent significant increase interest in mushrooms, especially wild growing species, is associated with the accumulation in their fruit bodies of high levels of trace elements (Kalač and Svaboda 2000; Isildak et al. 2004; Abdel-Azeem et al. 2007; Campos et al. 2009; Joshi et al. 2011; Severoglu et al. 2013; Mleczek et al. 2016a; Širić et al. 2016). Fruit bodies are able to accumulate variable contents of metals and often considerably higher levels of trace elements compared with vegetables, fruits, and agricultural crop plants (Kalač 2010; Huang et al. 2015; Širić et al. 2016), and also more than in animal tissue from the same ecosystems (Rakić et al. 2014). Mushrooms possess a very effective mechanism to accumulate trace elements from the environment (Doğan et al. 2006; Falandysz et al. 2008; Sesli et al. 2008; Severoglu et al. 2013), and the biosorption of these elements by fungal cells is a well-known phenomenon described in numerous studies (Sesli and Dalman 2006; Sesli et al. 2008; Mleczek et al. 2016a). Toxic metals (Hg) and/or metalloids (As) may be easily transported from polluted substrate to fruit bodies and eventually accumulate in human bodies (Falandysz and Borovička 2013; Mleczek et al. 2016c; Rzymski et al. 2016; Rubio et al. 2018). This trait is becoming more and more popular in terms of mycoremediation but absolutely not as regards the human nutrition (Li et al. 2017). For this reason, the growing consumption of both cultivated and—to a lesser extent—wild-growing mushroom species makes the control of the content of especially toxic elements in their fruit bodies a priority (Rashid et al. 2018). This issue is especially important in the case of medicinal mushrooms due to their aforementioned traits and positive influence for the human immune system (Agrawal and Dhanasekaran 2019).

Wild growing mushroom species are divided into edible, non-edible, and poisonous varieties; aboveground and wood-growing species; and those with different nutritional strategies (parasitic or saprobic). These differences have a substantial influence on the variations in metal accumulation in fruit bodies by up to one or even two orders of magnitude (Kalač 2010; Mleczek et al. 2016a). Different ecological types of mushroom species make them even more interesting in environmental investigations, particularly with respect to metal accumulation (Kalač 2001; Rakić et al. 2014; Širić et al. 2016).

In the case of wood-growing mushroom species, accumulation of trace elements is lower than in aboveground species (Gabriel et al. 1994; Petkovšek and Pokorny 2013), and is highly variable, even in the case of samples collected from the same place (Gabriel et al. 1994). Thus, wood-growing mushroom species, like epiphytic lichens, are good candidates for bioindicators of air pollution (Gabriel et al. 1994; Falandysz et al. 2007; Širić et al. 2016). Reports on the content of macro-, micro-, and trace elements in wood-growing mushroom species are limited and are generally focused on edible species (both wild and cultivated), and species with positive biological and medicinal effects, as summarized and presented in Table 1.

**Table 1:** Literature data on the content of selected elements (mg kg^−1^ DM) in some wood-growing mushroom species

Element	*A. mellea*^a^	*F. velutipes*^b^	*G. applanatum*^c^	*I. hispidus*^d^	*L. sulphureus*^e^	*P.igniarius*^f^	*S. crispa*^g^
Ba	0.8	–	–	–	0.9–4.38	–	–
Ca	–	–	2620	–	726–804	–	60
K	–	–	2180	–	27400–30500	–	20,280
Mg	1063.1	–	910	2.30	900–1012	–	720
Na	–	–	–	–	109.8–310	–	8313
P	–	–	–	–	4480–5300	–	4570
Ag	0.42–0.68	0.21	0.06	–	0.21–0.26	0.06	–
Al	4.8–192.9	7.0	7–744.8	–	18.25–53.96	–	–
As	0.1–0.33	0.39	0.09	–	0.33	–	0.57–1.03
B	1.60–3.018	0.1	0.383–6.2	–	0.70–16.4	–	–
Cd	0.5–20.3	0.63	0.12–0.82	–	0.06–3.21	nd	0.03–2.1
Co	0.06–1.173	0.15	0.09–0.744	–	0.03–1.136	–	0.23
Cr	0.12–930	0.09	0.06–17.46	10.20	0.09–58.3	84.5	0.11
Cu	0.091–45.6	22.70	9.4–52.1	30.00	2.1–35.2	28.9–66.1	1.86–6840
Fe	35–510	21.00	0.37–1400	114.00	28–362	900.00	36.62–46,120
Hg	0.19–0.91	–	–	–	–	–	–
Mn	0.081–55.59	5.20	15.5–52.7	53.00	2.4–30.7	30.9–81.1	5780
Ni	0.06–32.3	0.66	0.12–5.60	–	0.03–21.7	6.48–17.2	3.19
Pb	0.20–8.5	0.36	0.27–2.22	1.06	0.48–24.4	9.86	0.1–3.64
Se	0.36–0.79	0.42	0.06	191.00	0.09	–	–
Sn	3.803	–	3.531	–	4.525	–	–
Sr	0.6	1	5.2	–	1.3–6.95	–	–
Zn	0.11–76.8	43.00	21.09–101	194	29–130	145.00	2.34–75,180

^a^*A. mellea* (Demirbaş 2002; Isildak et al. 2004; Cocchi et al. 2006; Doğan et al. 2006; Sesli et al. 2008; Ouzouni et al. 2009; Durkan et al. 2011; Petkovšek and Pokorny 2013; Mleczek et al. 2016b; Širić et al. 2016; Wang et al. 2017)

^b^*F. velutipes* (Mleczek et al. 2016c)

^c^*G. applanatum* (Sesli and Dalman 2006; Durkan et al. 2011; Mleczek et al. 2016b; Raseta et al. 2016)

^d^*I. hispidus* (Wang and Hou 2011)

^e^*L. sulphureus* (Doğan et al. 2006; Sesli and Dalman 2006; Durkan et al. 2011; Kovács and Vetter 2015; Mleczek et al. 2016a)

^f^*P. igniarius* (Doğan et al. 2006; Sesli and Dalman 2006)

^g^*S. crispa* (Šlejkovec et al. 1997; Lee et al. 2013; Severoglu et al. 2013; Schlecht and Säumel 2015)

**Figure 1.**
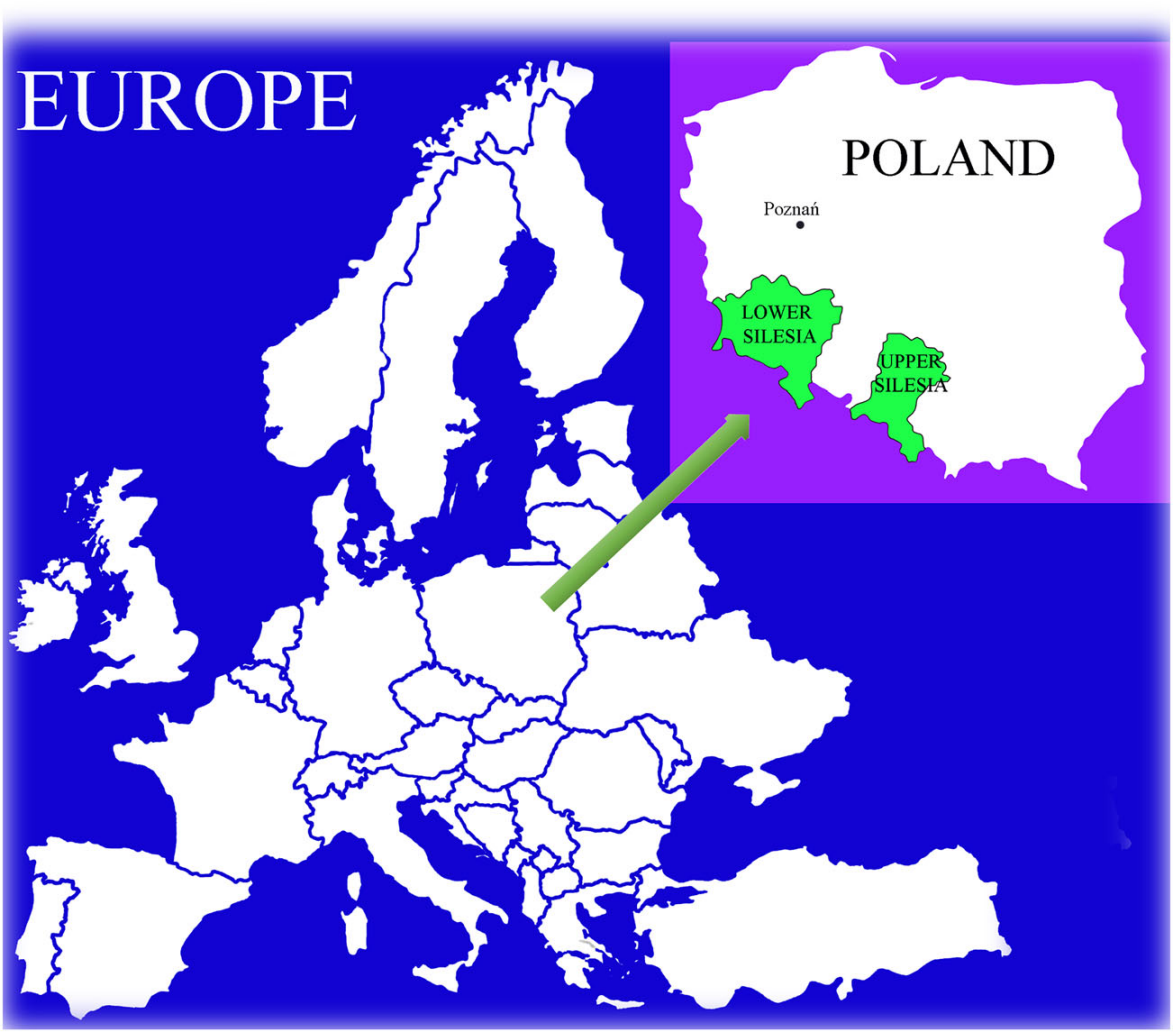
Localization of sampling sites of mushroom species collection

The objective of the present study was to compare 21 wild edible and non-edible wood-growing mushroom species collected from sites in Lower and Upper Silesia in Poland (Fig. 1) as regards their ability to accumulate all 27 of the detectable elements from among the 55 elements determined.

## Materials and methods

### Experimental materials

Twenty-one wood-growing edible and non-edible mushroom species belonging to the families Fomitopsidaceae (4), Ganodermataceae (3), Hericiaceae (1), Hymenochaetaceae (5), Meripilaceae (1), Phanerochaetaceae (1), Physalacriaceae (2), Polyporaceae (2), Sparassidaceae (1), and Strophariaceae (1) were compared in terms of their mineral composition of elements (Table 2).

**Table 2:** Characteristics of studied wood-growing mushroom species

Number	Wood-growing mushroom species	Family	Edibility	Nutritional strategy	Found on	Place
1.	*Armillaria mellea* (Vahl) P.Kumm.	Physalacriaceae	Edible	Parasitic	*Populus alba* L.	City park
2.	*Climacodon septentrionalis* (Fr.) P.Karst.	Phanerochaetaceae	Non-edible	Parasitic	*Acer pseudoplatanus* L.	Deciduous forest
3.	*Flammulina velutipes* (Curtis) Singer	Physalacriaceae	Edible	Saprobic/parasitic	*Acer negundo* L.	Deciduous forest
4.	*Fomitiporia robusta* (P.Karst.) Fiasson & Niemelä	Hymenochaetaceae	Non-edible	Parasitic	*Quercus robur* L.	Deciduous forest
5.	*Fomitopsis betulina* (Bull.) B.K. Cui, M.L. Han & Y.C. Dai	Fomitopsidaceae	Non-edible	Parasitic	*Betula pendula* Roth.	Deciduous forest
6.	*Fomitopsis pinicola* (Sw.) P. Karst.	Fomitopsidaceae	Non-edible, for medicinal uses	Parasitic	*Pinus sylvestris* L.	Pine forest
7.	*Fuscoporia wahlbergii* (Fr.) T.Wagner & M.Fisch.	Hymenochaetaceae	Non-edible	Parasitic	*Prunus domestica* L.	Old garden
8.	*Ganoderma appalanatum* (Pers.) Pat.	Ganodermataceae	Non-edible, for medicinal uses	Parasitic	*Fagus sylvatica* L.	Deciduous forest
9.	*Ganoderma pfeifferi* Bres.	Ganodermataceae	Non-edible, for medicinal uses	Parasitic	*Fagus sylvatica* L.	City park
10.	*Ganoderma resinaceum* Bound.	Ganodermataceae	Non-edible, for medicinal uses	Parasitic	*Fagus sylvatica* L.	Deciduous forest
11.	*Hemipholiota populnea* (Pers.) Knyper&Tjall.-Benk.	Strophariaceae	Non-edible	Parasitic	*Populus alba* L.	Deciduous forest
12.	*Hericium cirrhatum* (Pers.) Nicol.	Hericiaceae	Edible	Saprobic	*Fagus sylvatica* L.	Deciduous forest
13.	*Inonotus hispidus* (Bull.) P.Karst	Hymenochaetaceae	Non-edible, for medicinal uses	Parasitic	*Malus* sp.	Old garden
14.	*Ischnoderma resinosum* (Schard.) P.Karst.	Fomitopsidaceae	Non-edible	Saprobic	*Fagus sylvatica* L.	Deciduous forest
15.	*Laetiporus sulphureus* (Bull.) Murrill	Fomitopsidaceae	Edible, young fruiting bodies	Parasitic	*Robinia pseudoacacia* L.	City park
16.	*Meripilus giganteus* (Pers.) P.Karst.	Meripilaceae	Edible, young fruiting bodies	Saprobic/parasitic	*Acer platanoides* L.	City park
17.	*Phellinus igniarius* (L.) Quèll.	Hymenochaetaceae	Non-edible	Parasitic	*Populus nigra* L.	Deciduous forest
18.	*Phellinus pini* (Brot.) Pilát	Hymenochaetaceae	Non-edible	Parasitic	*Pinus sylvestris* L.	Pine forest
19.	*Polyporus septosporus* P.K. Buchanan & Ryvarden	Polyporaceae	Edible, young fruiting bodies	Parasitic/saprobic	*Fraxinus excelsior* L.	Deciduous forest
20.	*Sparassis crispa* (Wulfen) Fr.	Sparassidaceae	Edible	Parasitic	*Pinus sylvestris* L. (root)	Pine forest
21.	*Trametes versicolor* (L.) Lloyd	Polyporaceae	Non-edible, for medicinal uses	saprobic	*Carpinus betulus* L.	Deciduous forest

The majority of the studied mushroom species were collected from the same deciduous or pine forests located in Lower and Upper Silesia in Poland between 2013 and 2019. In the case of *A. mellea*, *G. pfeifferi*, *L. sulphureus*, and *M. giganteus*, fruit bodies were collected from a city park in Poznań, while *F. wahlbergii* and *I. hispidus* came from a mature garden in Poznań. Fruit bodies of mushroom species were collected between 2013 and 2019. The minimum amount of fruit bodies was 7 for the majority of mushroom species. On the other hand, some species were represented by 15 (*M. giganteus*), 16 (*T. versicolor*), 23 (*F. velutipes*), 25 (*A. mellea*), or 34 (*C. septentrionalis*) fruit bodies (Table 3). The reason for the significant differences in the number of fruit bodies of individual species was their limited occurrence in the studied forest areas.

**Table 3:** Characteristics of years and amount of fruit body samples collected

Number	Wood-growing mushroom species	1st year of collection	Amount of fruit bodies	2nd year of collection	Amount of fruit bodies	3rd year of collection	Amount of fruit bodies
1	*Armillaria mellea* (Vahl) P.Kumm.	2013	8	2014	12	2016	5
2	*Climacodon septentrionalis* (Fr.) P.Karst.	2013	12	2014	15	2018	7
3	*Flammulina velutipes* (Curtis) Singer	2014	8	2015	10	2016	5
4	*Fomitiporia robusta* (P.Karst.) Fiasson & Niemelä	2013	3	2014	2	2018	2
5	*Fomitopsis betulina* (Bull.) B.K. Cui, M.L. Han & Y.C. Dai	2013	3	2015	2	2017	2
6	*Fomitopsis pinicola* (Sw.) P. Karst.	2014	3	2015	3	2016	2
7	*Fuscoporia wahlbergii* (Fr.) T.Wagner & M.Fisch.	2013	3	2015	1	2019	3
8	*Ganoderma appalanatum* (Pers.) Pat.	2013	3	2014	3	2016	1
9	*Ganoderma pfeifferi* Bres.	2013	3	2015	2	2017	2
10	*Ganoderma resinaceum* Bound.	2013	2	2014	4	2016	1
11	*Hemipholiota populnea* (Pers.) Knyper&Tjall.-Benk.	2014	3	2015	3	2016	2
12	*Hericium cirrhatum* (Pers.) Nicol.	2013	3	2014	2	2016	2
13	*Inonotus hispidus* (Bull.) P.Karst	2013	3	2014	2	2017	2
14	*Ischnoderma resinosum* (Schard.) P.Karst.	2013	3	2014	4	2016	3
15	*Laetiporus sulphureus* (Bull.) Murrill	2014	2	2015	5	2017	2
16	*Meripilus giganteus* (Pers.) P.Karst.	2013	5	2014	7	2017	3
17	*Phellinus igniarius* (L.) Quèll.	2013	2	2015	2	2018	3
18	*Phellinus pini* (Brot.) Pilát	2013	3	2015	2	2018	2
19	*Polyporus septosporus* P.K. Buchanan & Ryvarden	2014	2	2015	4	2017	4
20	*Sparassis crispa* (Wulfen) Fr.	2013	3	2015	2	2017	2
21	*Trametes versicolor* (L.) Lloyd	2014	8	2015	4	2019	4

### Procedure

Samples were dried at 45 ± 1 °C for 120 h in an electric oven SLW 53 STD (Pol-Eko, Wodzisław Śląski, Poland) to determine the dry matter (dm) of samples which were then ground in a laboratory Cutting Mill SM 200 (Retsch GmbH, Haan, Germany). All fruit bodies of particular mushroom species collected from the same year were ground jointly to a powder fraction for 3 min. These materials were then homogenized with a B-400 ceramic knife homogenizer (Buchi Labortechnik AG, Flawil, Switzerland) within 3 min to obtain three representative samples that were digested using a microwave sample preparation system Mars 6 (CEM, Matthews, USA). Dry samples of mushroom were weighed (0.500 ± 0.001 g) using ME-T Analytical Balance (Metler Toledo, Columbus, USA) and digested by 5 mL of concentrated (65%) nitric acid (Merck, Darmstadt, Germany) in closed Teflon containers in the microwave sample preparation system. After digestion, samples were filtered using a paper filter: Qualitative Filter Papers, Grade 595: 4–7 μm (Whatman, Maidstone, UK), diluted with water (purified in an ion-exchange/reverse osmosis system (Millipore, Saint Luis, USA)) to a final volume of 10.0 mL. Each of the samples was analyzed in triplicate using the whole sample preparation procedure.

### Instruments

To analyze the mineral composition of the samples, an optical emission spectrometer with excitation by inductively coupled plasma Agilent 5110 ICP-OES (Agilent, Santa Clara, USA) was used. A simultaneous axial and radial view of plasma was allowed by the synchronous vertical dual view (SVDV). For multi-elemental determination, common conditions were applied: radio frequency (RF) power 1.2 kW, argon consumption 14.5 L min^−1^ (nebulizer gas flow 0.7 L min^−1^, auxiliary gas flow 1.0 L min^−1^, plasma gas flow 12.0 L min^−1^, polychromator purging gas 0.8 L min^−1^), viewing height for radial plasma observation 8 mm, and detector CCD (charge coupled device) temperature −40 °C; the signal was measured in three replicates by 5 s.

### Analytical method validation

Detection limits were determined at the level of 0.0X mg kg^−1^ dry weight (DW) for all elements determined (as 3-sigma criteria, Table S1). Uncertainty for the total analytical procedure (including sample preparation) was at the level of 20%. Traceability was checked by analysis of the reference materials CRM NCSDC 73349—bush branches and leaves (National Analysis Center for Iron & Steel, Beijing, China) and CRM CS-M-1—mushrooms (Institute of Nuclear Chemistry and Technique, Warsaw, Poland). The recovery (80–120%) was acceptable for most of the elements determined (Table S2). For uncertified elements, the recovery was defined using the standard addition method.

To avoid sample contamination, high (Suprapure) quality chemicals and water (18.2 MΩ) were used in preparation. The level of reagent blank was below detection limits for all elements determined. In addition, the control of analysis of a series of samples allowed any cross-contaminations to be avoided.

The content of 62 elements was determined in the studied mushroom species, but only 27 of them (Ag, Al, Ba, Ca, Cd, Cu, Fe, In, K, La, Mg, Mn, Na, Nd, Ni, P, Pb, Pr, Pt, Rh, Sr, Ti, Tm, V, Y, Zn, and Zr) were detectable in all 21 mushroom species. In the case of 28 elements (As, Au, B, Bi, Ce, Co, Cr, Dy, Er, Eu, Ga, Gd, Ge, Ho, Li, Lu, Mo, Os, Pd, Rb, Re, Sc, Se, Tb, Te, Tl, U, and Yb), their content in the fruit bodies of at least one species was below the limit of detection, while for the last 7 elements (Be, Hf, Ir, Ru, Sb, Sm, and Th), their content in all 21 mushroom species was below the limit of detection. For this reason, characteristics of mineral composition was performed for 55 elements, excluding the last mentioned group of metals.

### Statistical analysis

All the statistical analyses were performed using the agricole package (R). For a general comparison of the mean values of 55 particular elements in the studied mushroom species, the one-dimensional variable ANOVA and Tukey’s HSD (honestly significant difference) test were used. Moreover, for a graphical presentation of the relationships between the studied mushroom species with respect to the content of the 27 detectable elements in all the studied mushrooms, a principal component analysis (PCA) was performed (Morison 1990; Falniowski 2003). For the studied components, 41.1% (28.0 + 13.1) of the total variability was explained and differences between particular mushroom species were recorded. In addition, a heatmap with a hierarchical cluster analysis allowed a clear visualization of multidimensional data (mean contents of 27 detectable elements in particular mushroom species). Finally, to show which of the analyzed mushroom species was the most enriched with all detectable elements, the rank sum was calculated.

## Results

### Content of elements in mushroom species

The ranges for Ca, K, Mg, Na, and P in the whole population of all mushroom species were as follows: 15.4–470, 6580–44,600, 314–2150, 38.0–319, and 1100–15,500 mg kg^−1^ dm, respectively. The content of major elements (expressed on a dry mass basis each time) in the studied wood-growing mushroom species was significantly diverse (Table 4a). The highest mean content of Ca (318 mg kg^-1^) was determined in *P. igniarius* bodies, while the highest or a high K content in *F. velutipes*, *H. cirrhatum*, *I. hispidus*, and *P. pini* (32,000; 32,300; 30,700; and 37,700 mg kg^−1^, respectively). Also, in the case of Mg, Na, and P, more than one mushroom species was characterized by the highest mean content of these elements. The mean content of Mg over 1500 mg kg^−1^ was determined in *F. velutipes*, *F. betulina*, and *M. giganteus* (1770, 1570, and 1860 mg kg^−1^, respectively). *G. resinaceum* and *H. cirrhatum* were the two mushroom species most enriched with Na (254 and 283 mg kg^−1^, respectively), while the highest mean content of P was recorded in the aforementioned *M. giganteus* and also *P. pini* (10,700 and 13,900 mg kg^−1^, respectively). Generally, the content of trace elements was also diverse but for some of them, e.g., Ba, Ce, Eu, Ge, Ho, La, Ni, Pt, Rh, Tm, Y, or Zn, high similarity was also observed (Table 4b-e). Moreover, for selected trace elements (e.g., As, Au, Co, Cr, Ga, Li, Pd, Se), their mean content was over the limit of detection for some mushroom species only.

**Table 4:** Content of elements (mg kg^−1^ DM) in mushroom fruit bodies

a											
Mushroom species	**Ca**	**K**	**Mg**	**Na**	**P**	**Ag**	**Al**	**As**	**Au**	**B**	**Ba**
*A. mellea*	58.3^fg^	24,600^bcd^	485^def^	62.8^gh^	5300^d–g^	0.539^abc^	55.2^bc^	0.036^e^	0.168^c^	2.15^de^	4.07^d–g^
*C. septentrionalis*	45.9^g^	8530^g^	506^def^	147^c–f^	2700^fg^	0.127^bc^	43.6^b–e^	bDL	bDL	8.01^cde^	8.99^ab^
*F. robusta*	155^bcd^	12,100^efg^	661^def^	90.0^fgh^	5510^d–g^	0.674^a^	52.4^bcd^	0.889^cde^	bDL	14.0^bc^	6.03^b–e^
*F. velutipes*	24.6^g^	32,000^ab^	1770^a^	190^bcd^	8890^bcd^	0.043^c^	13.6^gh^	0.796^cde^	bDL	0.054^e^	2.01^fg^
*F. betulina*	26.1^g^	13,700^d–g^	1570^ab^	44.3^h^	3870^efg^	0.069^c^	11.6^h^	5.54^a^	0.023^d^	10.7^cd^	4.18^c–g^
*F. pinicola*	154^bcd^	8520^g^	738^def^	141^c–g^	1680^g^	0.184^abc^	18.4^e–h^	bDL	bDL	9.54^cde^	5.84^b–e^
*F. wahlbergii*	133^cde^	18,300^c–g^	1070^bcd^	51.6^h^	6550^c–f^	0.036^c^	7.18^h^	0.381^de^	bDL	23.5^b^	10.2^a^
*G. appalanatum*	119^c–f^	7470^g^	968^b–f^	72.0^fgh^	8850^bcd^	0.054^c^	10.9^h^	bDL	bDL	2.28^de^	1.74^g^
*G. pfeifferi*	163^bc^	16,800^c–g^	721^def^	70.2^fgh^	6560^c–f^	0.120^bc^	11.5^h^	1.29^c^	0.990^b^	5.02^cde^	5.76^b–f^
*G. resinaceum*	83.5^d–g^	13,000^d–g^	447^ef^	254^ab^	3190^efg^	0.076^c^	27.8^d–h^	1.12^cd^	bDL	bDL	4.46^c–g^
*H. populnea*	19.8^g^	31,400^b^	738^def^	198^bc^	5250^d–g^	0.163^abc^	3.97^h^	bDL	bDL	2.55^de^	1.98^fg^
*H. cirrhatum*	121^c–f^	32,300^ab^	609^def^	283^a^	6000^d–f^	0.458^abc^	14.2^fgh^	bDL	1.69^a^	7.44^cde^	3.04^efg^
*I. hispidus*	137^cde^	30,700^ab^	602^def^	111^d–h^	7150^b–e^	0.632^ab^	57.6^b^	bDL	bDL	14.0^bc^	7.94^abc^
*I. resinosum*	39.3^g^	14,300^c–g^	1020^b–e^	171^cde^	4430^efg^	0.135^bc^	15.0^fgh^	bDL	bDL	10.5^cd^	4.17^c–g^
*L. sulphureus*	82.7^efg^	20,700^b–f^	547^def^	99.4^e–h^	3780^efg^	0.073^c^	29.6^c–h^	0.394^de^	bDL	1.56^de^	2.99^efg^
*M. giganteus*	213^b^	22,100^b–e^	1860^a^	190^bcd^	10,700^abc^	0.340^abc^	89.3^a^	0.340^de^	bDL	11.2^cd^	2.76^efg^
*P. igniarius*	318^a^	14,900^c–g^	387^f^	85.2^fgh^	5290^d–g^	0.211^abc^	41.2^b–f^	bDL	bDL	67.3^a^	4.08^d–g^
*P. pini*	87.9^d–g^	37,700^a^	904^c–f^	143^c–g^	13,900^a^	0.087^c^	15.8^fgh^	bDL	bDL	2.74^de^	5.68^b–f^
*P. septosporus*	35.2^g^	26,100^abc^	1480^abc^	64.4^gh^	10,900^ab^	0.130^bc^	26.6^d–h^	bDL	bDL	7.62c^de^	4.98^c–g^
*S. crispa*	22.7^g^	18,800^c–g^	496^def^	73.0^fgh^	4950^d–g^	0.420^abc^	14.4^fgh^	2.57^b^	bDL	4.86^cde^	2.28^efg^
*T. versicolor*	140^cde^	9060^fg^	441^ef^	79.5^fgh^	1540^e^	0.126^bc^	40.1^b–g^	0.067^e^	bDL	6.02^cde^	6.91^a–d^
b											
Mushroom species	Bi	Cd	Ce	Co	Cr	Cu	Dy	Er	Eu	Fe	Ga
*A. mellea*	2.39^ab^	1.17^c–f^	bDL	0.034^d^	bDL	4.31^d^	0.069^b^	0.018^e^	0.103^a^	350^a^	0.327^a^
*C. septentrionalis*	0.367^h–k^	0.359^f^	0.246^bc^	0.028^d^	bDL	7.67^cd^	bDL	0.188^a^	0.074^a^	98.0^e–h^	bDL
*F. robusta*	2.15^abc^	2.17^cde^	bDL	bDL	0.401^a^	5.39^d^	0.023^de^	0.041^d^	0.066^ab^	62.0^g–j^	bDL
*F. velutipes*	0.694^f–k^	0.396^f^	0.147^cd^	0.043^d^	bDL	42.9^a^	0.036^cd^	bDL	bDL	160^bcd^	0.074^e–f^
*F. betulina*	1.15^d–h^	0.407^def^	bDL	0.032^d^	bDL	3.11^d^	0.025^de^	0.036^d^	bDL	51.5^hij^	bDL
*F. pinicola*	0.208^jk^	0.119^f^	0.152^cd^	bDL	0.024^c^	4.96^d^	bDL	0.043^d^	0.079^a^	100^e–h^	bDL
*F. wahlbergii*	1.41^c–f^	0.826^def^	0.026^d^	0.185^a^	bDL	13.6^bc^	bDL	0.129^bc^	0.029^bc^	19.5^j^	0.086^d–f^
*G. appalanatum*	0.446^g–k^	0.575^def^	0.012^d^	bDL	0.023^c^	7.65^cd^	bDL	0.002^f^	0.090^a^	104^d–h^	0.116^c–f^
*G. pfeifferi*	bDL	1.13^c–f^	bDL	0.122^b^	bDL	5.33^d^	0.065^b^	0.108^c^	0.106^a^	29.6^ij^	0.272^ab^
*G. resinaceum*	1.05^e–i^	1.13^c–f^	0.047^cd^	bDL	0.042^b^	10.8^bcd^	0.231^a^	0.038^d^	0.078^a^	80.9^f–i^	bDL
*H. populnea*	1.23^d–f^	9.46^a^	0.323^b^	bDL	0.048^b^	14.2^bc^	0.066^b^	bDL	bDL	37.1^ij^	0.172^b–e^
*H. cirrhatum*	1.97^a–d^	4.10^b^	bDL	bDL	bDL	10.3^cd^	0.005^e^	0.094^c^	0.109^a^	48.4^hij^	bDL
*I. hispidus*	0.284^ijk^	0.268^f^	0.152^cd^	bDL	bDL	9.57^cd^	0.050^bc^	0.023^d^	bDL	114^d–g^	bDL
*I. resinosum*	0.075^l^	0.831^def^	bDL	bDL	bDL	14.6^bc^	bDL	bDL	bDL	60.3^g–j^	0.039^f^
*L. sulphureus*	0.422^g–k^	0.615^def^	0.232^bc^	bDL	bDL	3.35^d^	bDL	0.226^a^	0.070^ab^	129^c–f^	0.202^a–d^
*M. giganteus*	0.851^e–j^	2.76^bc^	0.773^a^	bDL	0.048^b^	40.1^a^	0.034^cd^	bDL	bDL	208^b^	bDL
*P. igniarius*	0.635^f–k^	0.390^ef^	bDL	bDL	bDL	39.1^a^	bDL	0.147^b^	bDL	55.5^hij^	0.258^abc^
*P. pini*	1.65^b–e^	0.217^f^	bDL	0.155^ab^	bDL	3.08^d^	0.050^bc^	bDL	0.089^a^	177^bc^	bDL
*P. septosporus*	2.59^a^	0.558d^ef^	bDL	0.083^c^	bDL	18.5^b^	bDL	bDL	bDL	73.2^f–j^	0.328^a^
*S. crispa*	0.210^jk^	2.18^cd^	bDL	bDL	bDL	7.88^cd^	0.034^cd^	bDL	0.011^c^	80.0^f–i^	0.082^d–f^
*T. versicolor*	0.773^f–k^	0.570^def^	0.147^cd^	bDL	bDL	4.78^d^	bDL	0.012^e^	bDL	155^b–e^	bDL
c											
Mushroom species	Gd	Ge	Ho	In	La	Li	Lu	Mn	Mo	Nd	Ni
*A. mellea*	bDL	0.758^b–f^	bDL	5.90^abc^	0.039^ef^	bDL	0.011^c^	19.2^def^	bDL	0.271^ghi^	0.218^d^
*C. septentrionalis*	0.014^fg^	0.238^f^	0.116^abcde^	3.85^abc^	0.346^b^	0.759^a^	bDL	22.8^def^	3.48^a^	0.494^c–g^	0.273^d^
*F. robusta*	bDL	0.280^d–f^	0.097^bcde^	5.09a^bc^	0.149^c–f^	0.012^d^	bDL	38.4^bcd^	bDL	0.677^bcd^	0.312^d^
*F. velutipes*	0.049^cde^	0.929^b–f^	0.130^abcd^	4.27^abc^	0.109^c–f^	bDL	0.045^abc^	17.9^def^	0.639^b^	0.477^d–g^	7.089^a^
*F. betulina*	0.031^e–g^	1.32^bcd^	0.079^bcde^	4.22^abc^	0.090^c–f^	bDL	0.044^abc^	8.85^ef^	0.045^e^	0.277^ghi^	0.141^d^
*F. pinicola*	0.177^a^	bDL	0.168^abc^	3.74^abc^	0.152^cde^	bDL	bDL	8.91^ef^	bDL	0.284^ghi^	0.404^bcd^
*F. wahlbergii*	0.044^c–f^	0.812^b–f^	bDL	4.07^abc^	0.206^bc^	0.233^b^	0.076^ab^	31.0^cde^	0.17^de^	0.632^b–e^	1.45^b^
*G. appalanatum*	bDL	0.400^c–f^	0.132^abcd^	7.33^a^	0.057^def^	bDL	0.053^abc^	19.0^def^	0.478^c^	0.264^ghi^	0.467^bcd^
*G. pfeifferi*	0.011^g^	0.714^b–f^	0.020^e^	4.18^abc^	0.037^ef^	bDL	0.057^abc^	11.0^ef^	bDL	0.330^f–i^	0.143^d^
*G. resinaceum*	0.029^e–g^	0.975^b–f^	0.058^cde^	6.32^ab^	0.080^c–f^	0.095^c^	0.028^abc^	7.83^f^	bDL	0.437^d–h^	0.396^bcd^
*H. populnea*	bDL	0.434^c–f^	0.225^a^	2.51^bc^	0.009^f^	bDL	bDL	5.58^f^	0.383^c^	0.158^i^	0.098^d^
*H. cirrhatum*	bDL	1.14^b–e^	0.072^cde^	5.44^abc^	0.121^c–f^	bDL	0.015^bc^	10.9^ef^	0.053^f^	0.733^bc^	0.170^d^
*I. hispidus*	0.053^cd^	bDL	0.075^bcde^	5.61^abc^	0.129^c–f^	bDL	0.032^abc^	54.7^b^	0.033^f^	0.398^e–i^	0.790^bcd^
*I. resinosum*	0.034^d–g^	bDL	0.172^abc^	4.71^abc^	0.030^ef^	bDL	bDL	16.2^def^	bDL	0.212^hi^	0.533^bcd^
*L. sulphureus*	bDL	1.25^b–e^	0.122^abcd^	4.27^abc^	0.073^c–f^	bDL	0.088^a^	5.56^f^	bDL	0.336^f–i^	0.379^cd^
*M. giganteus*	0.145^b^	1.14^b–e^	bDL	4.82^abc^	0.568^a^	0.084^c^	bDL	89.0^a^	0.229^d^	0.997^a^	1.47^b^
*P. igniarius*	0.043^c–f^	3.01^a^	0.192^ab^	2.36^c^	0.186^cd^	bDL	0.024^abc^	59.1^b^	bDL	0.756^ab^	0.927^bc^
*P. pini*	bDL	1.42^bc^	0.148^abc^	3.26^bc^	0.072^c–f^	bDL	0.025^abc^	13.1^ef^	bDL	0.548^b–f^	0.051^d^
*P. septosporus*	0.078^c^	1.72^b^	0.108^abcde^	5.64^abc^	0.089^c–f^	0.099^c^	0.050^abc^	8.71^ef^	bDL	0.687^bcd^	0.135^d^
*S. crispa*	bDL	0.988^b–f^	bDL	6.08^abc^	0.040^ef^	bDL	0.070^abc^	21.4^def^	bDL	0.183^i^	0.171^d^
*T. versicolor*	0.040^d–f^	0.731^b–f^	0.223^a^	3.10^bc^	0.194^cd^	bDL	0.025^abc^	46.0^bc^	0.146^e^	0.454^d–h^	0.484^bcd^
d											
Mushroom species	Os	Pb	Pd	Pr	Pt	Rb	Re	Rh	Sc	Se	Sr
*A. mellea*	0.017^d^	0.389^e^	0.076^a^	1.52^b–f^	5.38^bcd^	6.12^e^	0.133^bcde^	0.589^a^	0.013^cd^	bDL	1.54^d^
*C. septentrionalis*	0.098^b^	4.38^a^	bDL	2.49^b^	3.97^b–e^	bDL	bDL	0.259^bcd^	0.018^bc^	bDL	8.27^bc^
*F. robusta*	bDL	1.18^cde^	bDL	1.17^c–h^	3.88^b–e^	bDL	bDL	0.250^bcd^	0.007^d^	0.898^b^	4.30^cd^
*F. velutipes*	0.043^cd^	1.29^cde^	bDL	0.076^i^	5.04^b–e^	0.902^f^	0.177^bcd^	0.192^cd^	0.026^b^	bDL	1.09^d^
*F. betulina*	0.085^bc^	1.59^cde^	bDL	0.383^ghi^	3.35^c–e^	bDL	0.238^ab^	0.255^bcd^	0.014^cd^	bDL	2.21^d^
*F. pinicola*	bDL	4.26^a^	bDL	0.975^c–i^	3.68^b–e^	bDL	0.071^cde^	0.187^cd^	0.014^cd^	bDL	39.8^a^
*F. wahlbergii*	0.034^d^	0.866^de^	bDL	4.41^a^	2.84^c–e^	0.656^f^	bDL	0.141^d^	bDL	1.14^a^	3.77^cd^
*G. appalanatum*	bDL	1.23^cde^	0.062^b^	1.56^b–e^	3.94^b–e^	bDL	0.087^cde^	0.324^bcd^	bDL	bDL	3.42^d^
*G. pfeifferi*	bDL	0.534^e^	bDL	1.92^bcd^	2.54^de^	bDL	0.366^a^	0.335^bcd^	0.013^bcd^	bDL	4.58^cd^
*G. resinaceum*	0.043^cd^	1.33^cde^	bDL	1.78^bcd^	5.69^bc^	bDL	bDL	0.261^bcd^	bDL	bDL	4.40^cd^
*H. populnea*	bDL	0.693^de^	bDL	0.459^f–i^	2.20^e^	81.2^a^	bDL	0.394^abc^	0.015^bc^	bDL	1.69^d^
*H. cirrhatum*	0.088^bc^	0.722^de^	bDL	0.415^ghi^	3.35^c–e^	70.8^b^	0.032^e^	0.185^cd^	bDL	0.106^d^	2.89^d^
*I. hispidus*	0.017^d^	2.36^bcd^	bDL	0.614^e–i^	3.32^c–e^	40.3^c^	bDL	0.120^d^	0.018^bc^	bDL	4.50^cd^
*I. resinosum*	0.024^d^	0.442^e^	0.029^c^	0.181^hi^	3.58^b–e^	62.3^b^	bDL	0.283^bcd^	0.008^d^	0.415^c^	1.50^d^
*L. sulphureus*	0.036^d^	1.32^cde^	bDL	1.58^b–e^	4.62^b–e^	bDL	bDL	0.136^d^	0.020^bc^	bDL	2.96^d^
*M. giganteus*	0.258^a^	4.28^a^	bDL	1.01^c–i^	10.1^a^	bDL	0.066^de^	0.335^bcd^	0.049^a^	bDL	10.5^b^
*P. igniarius*	0.095^b^	3.72^ab^	bDL	1.46^b–g^	5.05^b–e^	bDL	bDL	0.351^bcd^	0.015^bc^	bDL	12.7^b^
*P. pini*	bDL	1.77^cde^	bDL	2.04^bc^	6.30^b^	bDL	bDL	0.444^ab^	bDL	bDL	2.05^d^
*P. septosporus*	bDL	1.34^cde^	bDL	0.277^hi^	3.37^b–e^	5.64^e^	0.219^abc^	0.166^cd^	0.021^bc^	bDL	2.71^d^
*S. crispa*	bDL	2.88^abc^	bDL	0.174^hi^	3.53^b–e^	13.9^de^	0.154^bcd^	0.229^bcd^	0.022^bc^	bDL	1.00^d^
*T. versicolor*	bDL	1.79^cde^	bDL	0.904^d–i^	4.44^b–e^	22.4^d^	0.119^bcde^	0.125^d^	0.018^bc^	bDL	4.13^cd^
e											
Mushroom species	Tb	Te	Ti	Tl	Tm	U	V	Y	Yb	Zn	Zr
*A. mellea*	bDL	6.58^ab^	0.146^ef^	bDL	0.016^b^	0.223^e–g^	0.028^cd^	0.025^cd^	bDL	46.6^de^	0.017^gh^
*C. septentrionalis*	0.074^abc^	bDL	0.691^a–d^	1.29^b^	0.229^b^	0.853^ab^	0.133^b^	0.110^b^	bDL	74.0^cde^	0.081^bcd^
*F. robusta*	0.049^bc^	0.714^fg^	0.433^b–f^	bDL	0.075^b^	0.313^d–g^	0.083^bcd^	0.025^cd^	bDL	73.7^cde^	0.051^c–h^
*F. velutipes*	0.127^a^	0.906^fg^	0.688^a–d^	0.663^c^	0.035^b^	0.457^c–f^	0.072^bcd^	0.076^bc^	0.010^b^	66.1^cde^	0.129^a^
*F. betulina*	0.107^ab^	9.45^a^	0.230^d–f^	bDL	0.053^b^	0.463^c–f^	0.029^cd^	0.043^cd^	bDL	263^a^	0.088^abc^
*F. pinicola*	0.018^c^	0.918^fg^	0.786^abc^	bDL	0.106^b^	0.650^bcd^	0.020^d^	0.021^cd^	0.015^b^	67.1c^de^	0.043^d–h^
*F. wahlbergii*	0.063^abc^	1.22^efg^	0.058^f^	0.347^d^	0.174^b^	bDL	0.050^bcd^	0.024^cd^	bDL	116^c^	0.050^c–h^
*G. appalanatum*	0.012^d^	bDL	0.115^ef^	1.61^ab^	0.104^b^	0.290^d–g^	0.040^cd^	0.060^bcd^	bDL	55.2^cde^	0.032^e–h^
*G. pfeifferi*	0.045^bc^	9.69^a^	0.085^f^	1.34^b^	0.077^b^	bDL	0.026^cd^	0.020^cd^	bDL	55.0^cde^	0.089^abc^
*G. resinaceum*	bDL	1.01^efg^	0.405^b–f^	0.367^d^	0.041^b^	0.301^d–g^	0.027^cd^	0.035^cd^	bDL	51.7^de^	0.066^b–e^
*H. populnea*	bDL	5.50^bc^	0.036f	bDL	0.082^b^	0.500^b–f^	0.014^d^	0.050^cd^	bDL	78.1^cd^	0.026^e–h^
*H. cirrhatum*	bDL	1.10^efg^	0.192^def^	bDL	0.080^b^	0.791^abc^	0.049^bcd^	0.008^d^	bDL	51.3^de^	0.009^h^
*I. hispidus*	bDL	4.49^b–e^	0.920^ab^	2.05^a^	0.030^b^	0.389^def^	0.066^bcd^	0.031^cd^	0.012^b^	43.7^de^	0.064^b–f^
*I. resinosum*	bDL	4.08^b–f^	0.175^def^	bDL	0.044^b^	0.181^g^	0.005^d^	0.047^cd^	bDL	51.4^de^	0.023^f–h^
*L. sulphureus*	0.019^c^	2.76^c–g^	0.291^c–f^	1.18^b^	0.047^b^	0.194^fg^	0.110^bc^	0.043^cd^	0.013^b^	57.9^cde^	0.023^f–h^
*M. giganteus*	0.108^abc^	1.59^d–g^	0.990^a^	bDL	0.037^b^	1.04^a^	0.231^a^	0.221^a^	0.020^a^	181^b^	0.104^ab^
*P. igniarius*	0.032^bc^	0.427^g^	0.373^c–f^	bDL	0.103^b^	bDL	0.078^bcd^	0.063^bcd^	0.015^b^	14.3^e^	0.046^c–h^
*P. pini*	0.055^abc^	0.334^g^	0.109^ef^	bDL	0.083^b^	0.557^b–e^	0.011^d^	0.034^cd^	0.008^b^	86.6^cd^	0.033^e–h^
*P. septosporus*	0.032^bc^	2.47^c–g^	0.380^c–f^	bDL	0.083^b^	0.794^abc^	0.030^cd^	0.034^cd^	bDL	61.1^cde^	0.039^d–h^
*S. crispa*	0.062^abc^	4.79^bcd^	0.289^c–f^	1.37^b^	1.20^a^	0.461^c–f^	0.036^cd^	0.040^cd^	bDL	62.7^cde^	0.053^c–g^
*T. versicolor*	0.042^bc^	0.200^g^	0.629^a–e^	bDL	0.034^b^	0.430^c–f^	0.067^bcd^	0.042^cd^	0.011^b^	63.1^cde^	0.076^bcd^

*n* = 3; identical superscripts (a, b, c) denote non-significant differences between means in columns according to the post hoc Tukey’s HSD test

Ranges calculated for all the obtained results for trace elements with a content higher than the limit of detection in fruit bodies of all 21 mushroom species were as follows: 0.021–1.47 (Ag), 2.44–183 (Al), 0.721–11.3 (Ba), 0.036–10.4 (Cd), 1,15–47.8 (Cu), 17.8–353 (Fe), 1.88–9.44 (In), 0.011–0.654 (La), 2.06–103 (Mn), 0.137–1.15 (Nd), 0.046–8.70 (Ni), 0.361–4.93 (Pb), 0.066–4.85 (Pr), 1.90–11.6 (Pt), 0.038–0.629 (Rh), 0.627–43.8 (Sr), 0.031–1.14 (Ti), 0.013–2.34 (Tm), 0.010–0.266 (V), 0.010–0.255 (Y), 12.5–306 (Zn), and 0.010–0.131 (Zr) mg kg^−1^. In the case of the remaining elements that were not detectable in any of the studied wood-growing mushroom species, the following ranges were determined: 0.029–6.37 (As), 0.18–1.91 (Au), 0.034–75.9 (B), 0.060–3.25 (Bi), 0.010–0.891 (Ce), 0.025–0.199 (Co), 0.017–0.421 (Cr), 0.011–0.249 (Dy), 0.012–0.599 (Er), 0.023–0.123 (Eu), 0.034–0.428 (Ga), 0.010–0.221 (Gd), 0.224–3.389 (Ge), 0.019–0.345 (Ho), 0.011–0.855 (Li), 0.013–0.198 (Lu), 0.010–3.915 (Mo), 0.012–0.297 (Os), 0.025–0.082 (Pd), 0.600–90.3 (Rb), 0.028–0.408 (Re), 0.010–0.057 (Sc), 0.095–1.25 (Se), 0.010–0.201 (Tb), 0.012–11.0 (Te), 0.274–2.26 (Tl), 0.027–1.204 (U), and 0.013–0.023 (Yb) mg kg^−1^.

**Figure 2.**
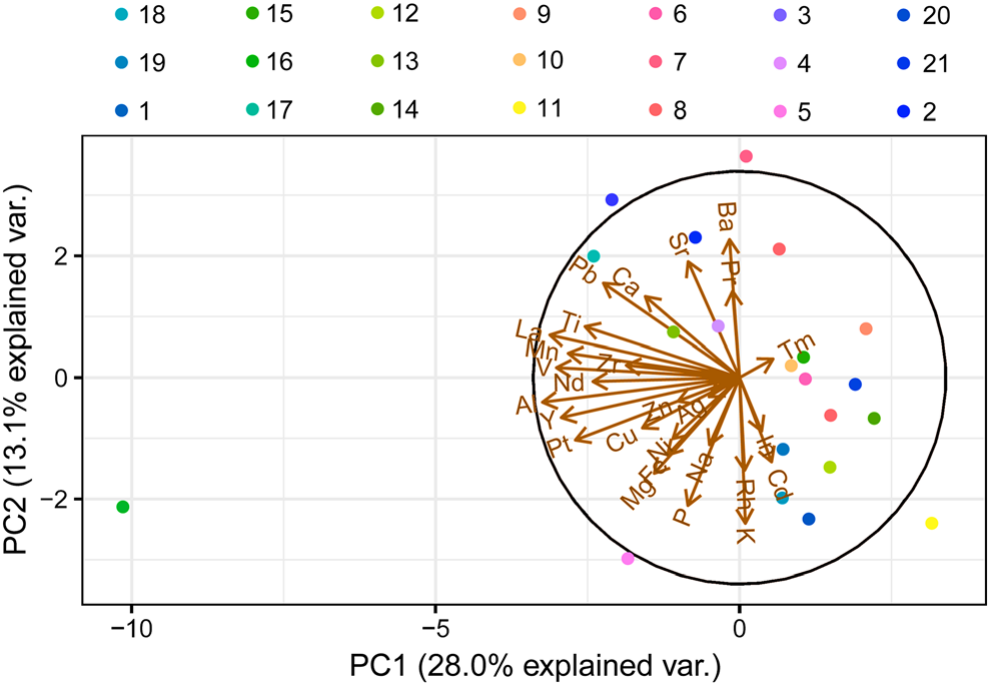
Graphical presentation of rank sum according to increase of the total content of elements in the studied mushroom species

The rank sum calculated for all 21 of the studied wood-growing mushroom species and all detectable major and trace elements showed that the fruit bodies of *M. giganteus*, which belongs to the Meripilaceae family, were the most enriched with all elements (Fig. 2). As *M. giganteus* is the only species of this family, it is difficult to state that it is exactly this factor that is responsible for the highest accumulation of the selected elements. The opposite situation was recorded for *P. igniarius*, *F. robusta*, *F. wahlbergii*, and *I. hispidus*, mushrooms belonging to the Hymenochaetaceae family, which were characterized as some of the most enriched. A similar situation was observed for *F. betulina*, *L. sulphureus*, and *I. resinosum* (Fomitopsidaceae family), which were less enriched with elements.

### Element composition—similarities and differences between mushrooms

**Figure 3.**
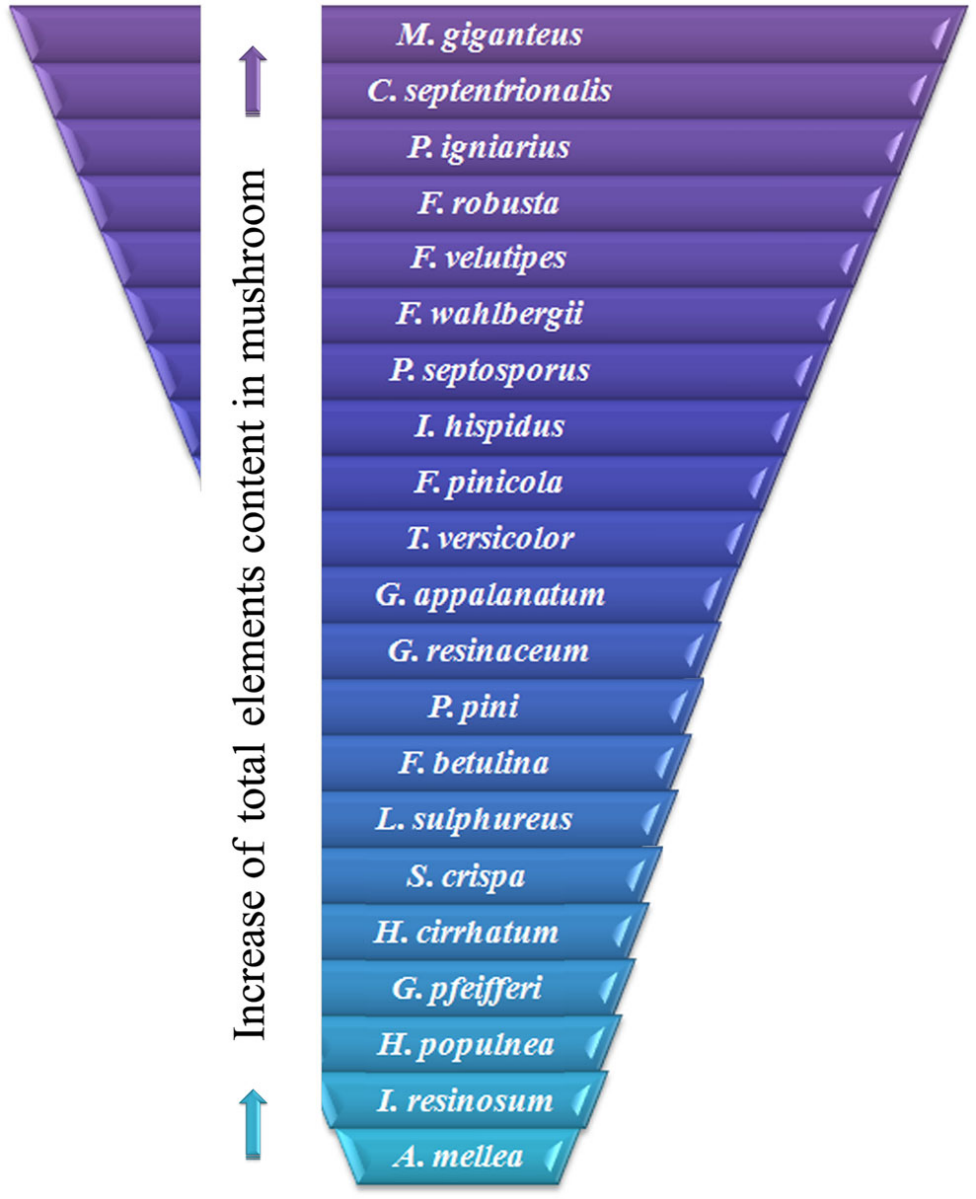
PCA for studied wood-growing mushroom species with regard to the content of all detectable elements

**Figure 4.**
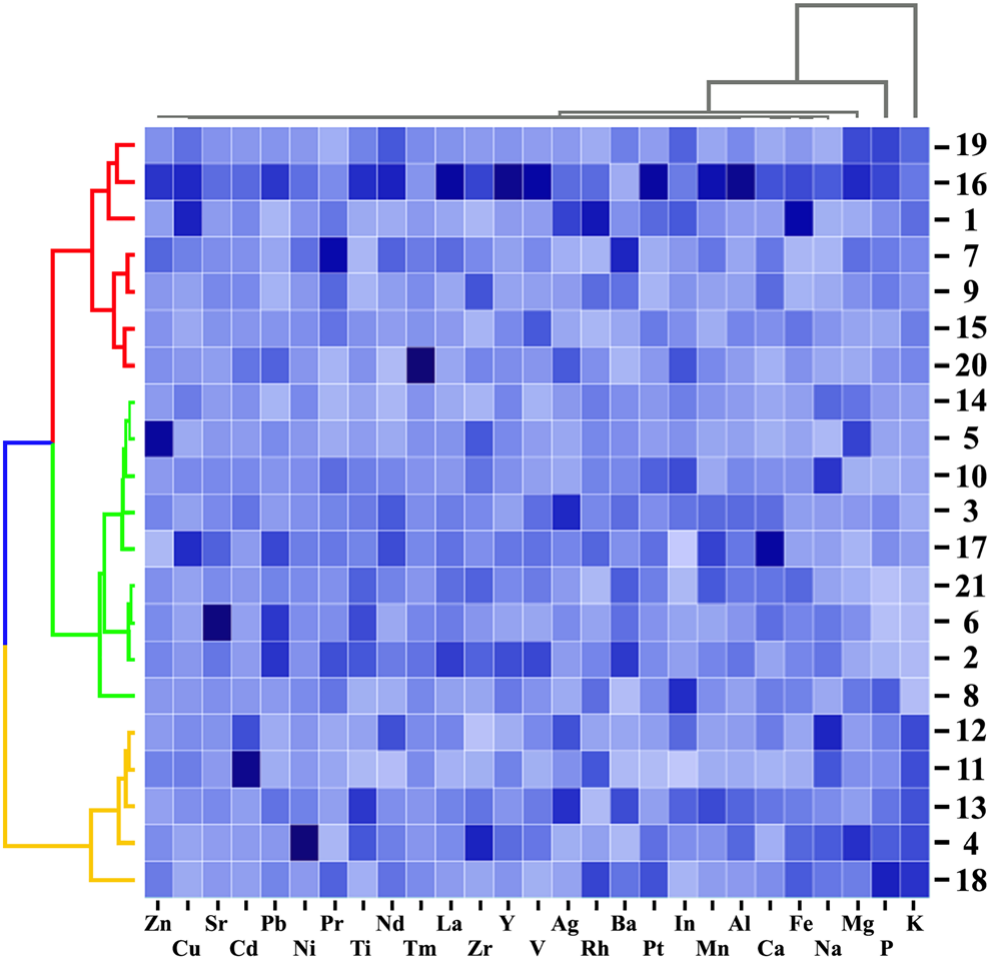
Correlation between 21studied mushroom species with regard to the content of detectable elements (heatmap) in mean values with presentation of a hierarchical tree plot

PCA was performed for all 21 mushroom species with regard to the content of all 27 detectable elements in each species (Fig. 3). The considerable distance of *M. giganteus* from the rest of the studied mushroom species and its high content of Al, Cu, La, Mg, Mn, Nd, P, Pt, Ti, V, or Y confirm that this species is the most enriched with the majority of detectable elements. The same was recorded for *H. populnea* which had the highest content of Cd (9.46 mg kg^−1^); *F. wahlbergii*, the most enriched with Ba and Pr (10.2 and 4.41 mg kg^−1^, respectively); and *P. igniarius* with the highest content of Ca. PCA for the wood-growing mushroom species explained 41.1% (28.0 + 13.1%) of total variability with clear differences between particular species and their ability to accumulate selected element(s) only. To better explain the similarities or differences between the studied mushrooms, a graphical presentation (heatmap) was performed together with hierarchical cluster dendrograms to group similar mushroom species with regard to the content of all 27 detectable elements (Fig. 4). *Meripilus giganteus*, characterized by the highest mean content of the majority of studied elements, was similar to *P. septosporus* and *A. mellea.* Generally, in terms of the content of all 27 elements, 3 separate groups of mushroom species can be extracted consisting of

The first group: *A. mellea*, *F. wahlbergii*, *G. pfeifferi*, *L. sulphureus, M. giganteus, P. septosporus,* and *S. crispa*;

The second group: *C. septentrionalis, F. robusta*, *F. betulina*, *F. pinicola*, *G. appalanatum, G. resinaceum*, *I. resinosum*, *P. igniarius,* and *T. versicolor*;

The third group: *F. velutipes, H. cirrhatum*, *H. populnea*, *I. hispidus*, and *P. pini*.

In spite of the similarities between mushroom species belonging to the same group, differences in the content of particular elements in their fruit bodies may observed. An example is clearly higher content of Ag, Fe, and Rh in *A. mellea* than was found in *M. giganteus* fruit bodies or the higher content of Sr in *F. pinicola* compared with *T. versicolor* despite their high similarity in the content of all elements jointly.

## Discussion

Many studies monitor the content of elements in the fruit bodies of edible wild growing and/or cultivated mushroom species (Rzymski et al. 2017; Kalač 2019). However, the data on element content in wood-growing mushrooms are relatively limited when compared with the amount available for aboveground species. Wood-growing mushrooms do not play an important role in consumption, many of them are non-edible and only a few species are used as medicinal mushrooms (Sokół et al. 2015). This is the most likely reason why their wider mineral composition has been studied for only a few species (Strapáč and Baranová 2016).

In the case of aboveground mushrooms, efficiency of element accumulation depends on both their concentration in substrate (significance of bedrock geochemistry) and mushroom species (Wang et al. 2015; Plassard et al. 2011). Mushrooms are able to effectively accumulate elements (more effectively than vascular plants) even at relatively low concentrations in the medium (Falandysz and Borovicka 2013). It is necessary to know whether their growth is tree–mushroom symbiosis dependent. For this reason, it is possible that the efficiency of element accumulation in the studied mushroom fruit bodies was just a response to their content in wood. Trees are promising plants for dendroremediation purposes, but are highly diverse in terms of their efficiency for phytoextraction of elements from soil (Mleczek et al. 2018). These differences are also observed in the site of element deposition within a plant; therefore, the occurrence of wood-growing mushrooms at a certain height on a tree may determine higher or lower accumulation of specific elements or the limitation of others (Mleczek et al. 2016c).

It is worth underlining that some of the observed similarities between the studied mushroom species were previously described by Mleczek et al. (2016a) who compared 10 wood-growing mushroom species, e.g., *A. mellea*, *F. velutipes*, *G. applanatum*, and *L. sulphureus.* The authors noted a similarity between all the aforementioned mushroom species with respect to Pb, Re, and Zn content, the highest content of Fe in *A. mellea*, and the highest content of As, Cu, and Ni in *F. velutipes* with the lowest content of B in this mushroom. In addition, a similarity between the content of Ag and Al in *G. applanatum* and *L. sulphureus* with a higher content in *A. mellea* was also recorded, which suggests that the selected mushroom species are more effective accumulators than others. The relationships described in the present paper may suggest that the belonging of certain species of mushrooms to a particular family may be associated with greater or less metal accumulation. On the other hand, the example of the highly diverse *F. velutipes* and *A. mellea* (both belong to Physalacriaceae family) indicates that it is not only family but also tree species that may influence the content of elements in fruit bodies. Similarities or differences in element accumulation between some mushroom species of the same family have been described in numerous papers (Wang et al. 2015). As in our paper, Chemghom et al. (2010) reported the highest content of Ca and a high content of Mn in *P. igniarius*, *Flammulina velutipes* grown on *Acer negundo*, and *A. mellea* on *Populus alba* L.; what is especially important is that *Acer* is generally characterized by more effective phytoextraction of elements from soil than *Populus* (Tangahu et al. 2011; Mleczek et al. 2017a).

One of the most effective species in accumulating elements was *M. giganteus*, being earlier characterized by Kalyoncu et al. (2010) with a higher content of Na and Zn than in *A. mellea* and *S. crispa*, or a similar content of K to that of *A. mellea* and higher than that found in *S. crispa*. In addition, a higher content of Mg in *M. giganteus* compared with some other mushroom species was described by Yildiz et al. (2019) and Fe reported by Karaman and Matavulj (2005). The ability of hyperaccumulators and also mushroom species such as *M. giganteus* to accumulate some elements (e.g., Mg, Cu, La, Zn) with the exclusion of others (e.g., Co, Lu, Rb, Se, or Tl) may be an effect of a specific strategy to discriminate homologue elements (Falandysz and Borovicka 2013). *Armillaria mellea* was characterized by a comparable content of Ca and Pb with that of Romanian samples, while the content of Ag, As, B, Cd, Cu, Li, Mn, Ni, Se, and Tl was lower (Zavastin et al. 2018). Fruit bodies of this mushroom species from West Macedonia and Epirus contained higher amounts of Mg, Cd, Co, Cu, Cr, Fe, Mn, Ni, Pb, and Zn (Ouzouni et al. 2009). Širić et al. (2016) analyzed this lignicoluous saprophyte collected from the Nature Park Medvednica (Croatia) with a higher content of Cu and Zn and a lower content of Fe than in our paper. However, the screening of samples from Hungary pointed to a higher content of Ba, Ca, Cd, Co, K, Mg, Na, Ni, P, and Sr, a lower one of Cu and Fe, and a similar content of Zn (Kovács and Vetter 2015), while Šlejkovec et al. (1997) indicated a lower level of As in *S. crispa*, and Severoglu et al. (2013) found a lower content of Cd, Cu, Fe, and Zn, and a higher level of Co, Cr, Ni, and Pb. Doğan et al. (2006) studied 32 mushroom species, among others: *A. mellea*, *L. sulphureus*, *P. igniarius, and T. versicolor* with regard to the content of Ag, Cd, Cr, Cu, Mn, Ni, and Pb. The mean content of Cr, Cu, Ni, and Pb was higher, while Ag, Cd, and Mn was comparable with that of the *A. mellea* fruit bodies described in this and our studies. With the exception of Cd, whose mean content was similar (0.68 and 0.615 mg kg^−1^), the content of the other determined metals was higher in *L. sulphureus* fruit bodies. The mean content of Cr, Ni, and Pb in *P. igniarius* (84.5, 6.48, and 9.86 mg kg^−1^, respectively) was higher than in our results (bDL; 0.927 and 3.72 mg kg^−1^, respectively), while the content of the other metals studied by Doğan et al. (2006) was lower. In addition, Ag, Cr, Cu, Mn, and Ni content was higher, and Cd and Pb lower in the study of Doğan et al. (2006) on *T. versicolor*. Not all papers provide enough information about the tree species on which the wood-growing mushroom species grow. A good example for discussion is the same study of Doğan et al. (2006). *Armillaria mellea*, growing on poplar trees in both studies, is able to accumulate a relatively high amount of selected elements only (Laureysens et al. 2004), which can explain their higher level in the mushroom body.

*Laetiporus sulphureus* grows on *Robinia pseudoacacia* L. and willow trees. In spite of numerous literature data that point to the highly diverse efficiency of element phytoextraction by willow taxa, it is very difficult to decide whether the growth of *L. sulphureus* was a key factor affecting the higher or lower content of elements in mushrooms (Mleczek et al. 2017b; Nirola et al. 2015; Yang et al. 2014). *Phellinus igniarius* grows on cedar and poplar trees, which may explain the significantly higher content of Cr and lower amount of Cd in the mushrooms studied by Doğan et al. (2006) (Onder and Dursun 2006). Furthermore, in the case of *T. versicolor*, differences in a higher/lower content in the fruit bodies of this mushroom may be explained by its growth on *Carpinus betulus* L. or poplar trees (Kaszala et al. 2003; Bilek et al. 2016).

The content of Ca, Cd, Cr, Cu, Mn, Ni, and Pb for *G. appalanatum* was lower, while that of Fe, K, Mg, and Zn was higher when compared with the results of the studied mushroom species from Serbia (Raseta et al. 2016). Wang and Hou (2011) studied *I. hispidus* and obtained results for Fe and Mn that were similar to our studies. In addition, a higher content of Cr, Se, and Zn but a lower content of Cu, Mg, and Pb was observed. Generally, the analyzed fruit bodies were characterized by a lower content of Ag, Ca, Cd, Cu, Cr, Mn, Ni, P, Pb, and Zn, when compared with samples originated from Turkey (Demirbaş 2002; Isildak et al. 2004), Cr and Pb from China (Wang et al. 2017) and also As, Cd, Pb, and Se, than mushrooms collected from Italy (Cocchi et al. 2006). The differences between the results presented in the present paper and the authors of other studies confirm a significant variability in the accumulation of elements by the same species of mushrooms depending on their place of growth. This suggests that the uptake of elements by wood-growing mushrooms is a matter that depends not only on the species but primarily on the species of tree on which this fungus resides, as well as the type of soil (concentration of bioavailable elements) where aboveground mushroom species grow.

## Conclusions

Wood-growing mushroom species play an important role in forest ecosystems because of their symbiosis with trees or the ability of these saprotrophic organisms to decompose dead organic matter. Their cultural significance, as well as the possibility to use them in the industry (pigments, bioactive compounds in pharmacology), makes them an interesting subject of research. In addition, their chemical purity plays a significant role in terms of their further use for practical purposes. The obtained results indicated a clearly differentiated content of individual as well as the sum of examined elements in particular mushroom species. Due to their growth on different species of trees and shrubs growing in soils with different chemical composition, it is difficult to clearly identify a higher or lower ability to accumulate particular elements. The analysis of both common and rarely occurring species of forest mushrooms over a 7-year period (2013–2019) has shown that similarly to wild-growing aboveground mushroom species, wood-growing mushrooms can effectively absorb major and trace elements.

## Electronic supplementary material

ESM 1 (DOCX 22 kb)
