# Supplementary material for: Mineral composition of elements in wood-growing mushroom species collected from of two regions of Poland
Source: Environ Sci Pollut Res Int. 2020 Sep 17;28(4):4430–42. doi: 10.1007/s11356-020-10788-y (PMC7835311; doi:10.1007/s11356-020-10788-y)
Supplement: Supplementary file 1 — (DOCX 22 kb) [file 11356_2020_10788_MOESM1_ESM.docx]

**Supplementary data** to

**"Mineral composition of elements in wood-growing mushroom species collected from of two regions of Poland"** by Mleczek et al.

Table S1. Validation data for elemental analysis by ICP-OES

| Element | wavelength | DL | uncertainty | Element | wavelength | DL | uncertainty |
| --- | --- | --- | --- | --- | --- | --- | --- |
|  | nm | mg kg^-1^ | % |  | nm | mg kg^-1^ | % |
| Ag | 328.068 | 0.017 | 10.0 | Mg | 279.553 | 0.016 | 9.7 |
| Al | 396.152 | 0.011 | 2.9 | Mn | 257.610 | 0.004 | 1.8 |
| As | 188.980 | 0.024 | 14.6 | Mo | 202.032 | 0.088 | 3.1 |
| Au | 197.742 | 0.018 | 18.0 | Na | 588.995 | 0.052 | 6.7 |
| B | 249.772 | 0.094 | 3.4 | Nd | 406.108 | 0.024 | 6.2 |
| Ba | 455.403 | 0.014 | 9.0 | Ni | 231.604 | 0.018 | 8.0 |
| Bi | 223.061 | 0.013 | 4.1 | P | 213.618 | 0.640 | 10.7 |
| Ca | 422.673 | 0.072 | 2.8 | Pb | 220.353 | 0.046 | 4.2 |
| Cd | 214.439 | 0.005 | 10.1 | Pd | 340.458 | 0.050 | 9.0 |
| Ce | 446.021 | 0.046 | 13.6 | Pr | 417.939 | 0.064 | 9.8 |
| Co | 238.892 | 0.006 | 1.6 | Pt | 203.646 | 0.042 | 29.8 |
| Cr | 267.716 | 0.007 | 4.0 | Re | 197.248 | 0.064 | 5.0 |
| Cu | 327.395 | 0.005 | 9.6 | Rh | 343.488 | 0.070 | 13.3 |
| Dy | 364.540 | 0.046 | 6.7 | Ru | 240.272 | 0.042 | 9.4 |
| Er | 349.910 | 0.036 | 7.9 | Sb | 206.834 | 0.024 | 14.0 |
| Eu | 420.504 | 0.068 | 3.0 | Sc | 361.383 | 0.048 | 3.0 |
| Fe | 238.204 | 0.017 | 1.6 | Se | 196.026 | 0.022 | 16.4 |
| Ga | 294.363 | 0.019 | 3.4 | Sm | 442.434 | 0.052 | 9.8 |
| Gd | 342.246 | 0.068 | 6.1 | Sn | 283.998 | 0.013 | 12.3 |
| Ge | 209.426 | 0.078 | 2.2 | Sr | 460.733 | 0.018 | 5.3 |
| Hg | 194.164 | 0.046 | 14.6 | Te | 214.282 | 0.022 | 5.5 |
| Ho | 348.484 | 0.062 | 11.9 | Tl | 190.794 | 0.048 | 7.3 |
| In | 230.606 | 0.050 | 8.0 | Tm | 336.261 | 0.062 | 7.6 |
| Ir | 205.116 | 0.019 | 6.5 | V | 292.401 | 0.036 | 6.2 |
| K | 766.491 | 0.068 | 1.3 | Y | 361.104 | 0.062 | 6.2 |
| La | 398.852 | 0.017 | 6.8 | Yb | 328.937 | 0.028 | 3.8 |
| Li | 670.783 | 0.009 | 16.6 | Zn | 213.857 | 0.004 | 3.5 |
| Lu | 307.760 | 0.062 | 2.9 |  |  |  |  |

Table S2. Results of certified reference materials analysis

| **Element** | CRM CS-M-1  mushroom | | | CRM NCSDC  plant | | |
| --- | --- | --- | --- | --- | --- | --- |
|  | certified | determined | recovery | certified | determined | recovery |
|  | [mg kg^-1^] | [mg kg^-1^] | [%] | [mg kg^-1^] | [mg kg^-1^] | [%] |
| Ag | - | <0.01 | - | 0.049 | 0.05 | 102 |
| Al | - | 72 | - | 2000 | 1678 | 84 |
| As | 0.344 | 0.31 | 90 | 1.25 | 1.01 | 81 |
| Au | - | 0.81 | - | - | 0.91 | - |
| B | - | 2.3 | - | 38 | 36 | 95 |
| Ba | - | 2.1 | - | 18 | 13 | 74 |
| Bi | - | <0.01 | - | 0.023 | 0.02 | 87 |
| Ca | - | 301 | - | 16800 | 16098 | 96 |
| Cd | 0.273 | 0.25 | 92 | 0.38 | 0.41 | 108 |
| Ce | - | 0.32 | - | 2.2 | 2.4 | 109 |
| Co | - | 0.11 | - | 0.41 | 0.38 | 93 |
| Cr | - | 0.48 | - | 2.6 | 2.1 | 81 |
| Cu | - | 7.1 | - | 6.6 | 6.1 | 92 |
| Dy | - | <0.01 | - | 0.13 | 0.11 | 85 |
| Er | - | 1.4 | - | - | 4 | - |
| Eu | - | <0.01 | - | 0.039 | 0.04 | 103 |
| Fe | - | 104 | - | 1070 | 943 | 88 |
| Ga | - | <0.01 | - | - | <0.01 | - |
| Gd | - | <0.01 | - | 0.19 | 0.21 | 111 |
| Ge | - | <0.01 | - | - | <0.01 | - |
| Ho | - | <0.01 | - | 0.033 | 0.03 | 91 |
| In | - | 0.87 | - | - | <0.01 | - |
| Ir | - | 0.62 | - | - | 1.1 | - |
| K | - | 14874 | - | 9200 | 8712 | 95 |
| La | - | 0.12 | - | 1.25 | 1.10 | 88 |
| Li | - | 0.09 | - | 2.6 | 2.9 | 112 |
| Lu | - | <0.01 | - | 0.011 | <0.01 | - |
| Mg | - | 634 | - | 4800 | 4320 | 90 |
| Mn | - | 12 | - | 61 | 56 | 92 |
| Mo | - | 13 | - | 0.28 | 0.23 | 82 |
| Na | - | 243 | - | 19600 | 17456 | 89 |
| Nd | - | 0.55 | - | 1 | 1.1 | 110 |
| Ni | - | 0.33 | - | 1.7 | 1.4 | 82 |
| Pb | 0.476 | 0.45 | 95 | 47 | 44 | 94 |
| Pd | - | <0.01 | - | - | <0.01 | - |
| Pr | - | 0.11 | - | 0.24 | 0.22 | 92 |
| Pt | - | 0.90 | - | - | 0.40 | - |
| Re | - | 0.12 | - | - | 0.20 | - |
| Rh | - | <0.01 | - | - | <0.01 | - |
| Ru | - | <0.01 | - | - | 0.11 | - |
| Sb | - | 0.19 | - | 0.095 | 0.08 | 84 |
| Sc | - | <0.01 | - | - | 0.22 | - |
| Se | 1.37 | 1.5 | 106 | - | 0.21 | - |
| Sm | - | 0.13 | - | 0.19 | <0.01 | - |
| Sr | - | 1.1 | - | 246 | 254 | 103 |
| Te | - | <0.01 | - | - | 0.34 | - |
| Tl | - | <0.01 | - | - | <0.01 | - |
| Tm | - | <0.01 | - | - | 0.11 | - |
| Y | - | <0.01 | - | 0.68 | 0.61 | 90 |
| Yb | - | <0.01 | - | 0.063 | 0.07 | 111 |
| Zn | 60.9 | 62 | 102 | 55 | 58 | 105 |
